# Modern applications of cross-classified random effects models in social and behavioral research: Illustration with R package PLmixed

**DOI:** 10.3389/fpsyg.2022.976964

**Published:** 2022-10-24

**Authors:** Sijia Huang, Minjeong Jeon

**Affiliations:** ^1^School of Education, Indiana University Bloomington, Bloomington, IN, United States; ^2^School of Education and Information Studies, University of California, Los Angeles, Los Angeles, CA, United States

**Keywords:** cross-classified data, multilevel model, item response theory, multitrait-multimethod, network analysis, generalizability theory, PLmixed

## Abstract

Cross-classified random effects models (CCREMs) have been developed for appropriately analyzing data with a cross-classified structure. Despite its flexibility and the prevalence of cross-classified data in social and behavioral research, CCREMs have been under-utilized in applied research. In this article, we present CCREMs as a general and flexible modeling framework, and present a wide range of existing models designed for different purposes as special instances of CCREMs. We also introduce several less well-known applications of CCREMs. The flexibility of CCREMs allows these models to be easily extended to address substantive questions. We use the free R package **PLmixed** to illustrate the estimation of these models, and show how the general language of the CCREM framework can be translated into specific modeling contexts.

## 1. Introduction

Data in social and behavioral studies often show multilevel structures (Bryk and Raudenbush, 1992). The multilevel structure may be purely hierarchical (or nested). In a purely hierarchical structure, lower-level units belong to one and only one higher-level unit. A canonical example of strictly nested data is found where students (at level 1) are nested within classrooms (at level 2), and classrooms are nested within schools (at level 3). A pure hierarchy as such may no longer hold when lower-level units belong to two or more types of higher-level units at the same level. This kind of situation yields a so-called cross-classified structure. An example of cross-classified data can be found where students (at level 1) are nested within schools and neighborhoods simultaneously (at level 2). Students who live in the same neighborhoods may not attend the same schools, and students who attend the same schools may be drawn from different neighborhoods; therefore the schools and neighborhoods are not nested but crossed. If lower-level units belong to two or more higher-level units of the same type, the data show a multiple membership structure, rather than crossed; for example, students attend more than one classroom (multiple classroom membership) or more than one middle school (multiple school membership). We will focus on cross-classified structures, not multiple membership structures, in this paper.

Standard regression models designed for single-level data typically assume that units of analysis are independent of each other. This independence assumption is violated when the data show multilevel structures due to the clustering of units, i.e., dependence among the units within the same clusters. Hierarchical linear models (HLM; Bryk and Raudenbush, 1992), also called multilevel models, have been developed to address such lack of independence and account for dependence among units within the clusters where the units are nested. To accommodate more complex dependence structures due to cross-classified clustering in data, multilevel models have been extended to cross-classified random effects models (CCREMs; Raudenbush, 1993; Goldstein, 1994; Rasbash and Goldstein, 1994). In applied settings, researchers may fail to recognize the cross-classified structure of the data being analyzed, neglecting some or all crossed factors in data analysis. However, studies have shown that ignoring crossed factors can bias variance estimates and standard errors of the regression coefficients associated with predictors (Meyers and Beretvas, 2006; Luo and Kwok, 2009; Ye and Daniel, 2017).

To promote the use of CCREMs in applied research, we aim to introduce modern applications of CCREMs that may be less well-known in the literature. In particular, we will present several existing statistical/psychometric models as applications of CCREMs. Understanding these models as CCREMs can be beneficial; the flexibility of CCREMs allows researchers to extend these models to address important substantive issues by incorporating complex multilevel/cross-classified data structures, covariates, and other model structures. In addition, researchers can estimate these models and their extensions using existing software packages available for CCREMs. This means that researchers may not need to learn different software packages designed for specific models separately every time they need them.

We will illustrate the estimation of the discussed CCREM applications with a freely available R package, **PLmixed** (Jeon and Rockwood, 2018). **PLmixed** is a flexible R package designed to estimate models with nested and/or crossed random effects. It can estimate standard CCREMs as well as extended CCREMs with factor structures (e.g., factor loadings). The latter type, cross-classified models combined with measurement models, may not be estimable with other widely-used R packages, such as **lavaan** (Rosseel, 2012) and **mirt** (Chalmers, 2012). Further, **PLmixed** extends the popular R package **lme4** (Bates et al., 2015), so researchers who are familiar with **lme4** syntax can use **PLmixed** with minimal learning efforts. For each of the applications, we provide the **PLmixed** syntax and an example dataset (in the manuscript and in the supplement), so that readers can easily apply and utilize the discussed models in their own research.

The remainder of this paper is organized as follows. In Section 2, we give a context to establish the notation of CCREMs. In Section 3, we briefly review available software options for CCREM estimation, and provide an overview of the **PLmixed** package. In Section 4, we present several statistical/psychometric models as special cases of CCREMs, and then present a couple of other less well-known, but interesting applications of CCREMs. Finally, in Section 5, we conclude the paper with a summary and a discussion on limitations and future directions.

## 2. Model

### 2.1. Setting

To set up the notation, we suppose a researcher wants to study the effects of elementary schools and neighborhoods on students' math performance, where the math performance was measured based on standardized test scores (continuous) or pass/fail evaluations (binary). The researcher also wants to explore how some characteristics of schools (e.g., public vs private), neighborhoods (e.g., residential income deprivation scores), and students (e.g., student age and race/ethnicity) may be related to the student performance measure. In this case students, the level-1 units, are cross-classified by two different level-2 units, schools and neighborhoods.

### 2.2. General formulation

Let *y*_*ijk*_ denote the math performance of student *i* (*i* = 1, …, *I*) who goes to school *j*(*j* = 1, …, *J*) and lives in neighborhood *k*(*k* = 1, …, *K*). *I*, *J* and *K* are the total numbers of students, schools and neighborhoods, respectively. We specify a CCREM for *y*_*ijk*_ as follows:

(1) $$\begin{array}{l} g\left(E\left(y_{ijk}|u_{j},u_{k}\right)\right)={\text{X}}_{ijk}\beta +u_{j}+u_{k}, \end{array}$$

where *g*(·) is a link function that transforms the linear predictor on the right-hand side to the conditional expectation *E*(*y*_*ijk*_|*u*_*j*_, *u*_*k*_). Using a link function allows us to accommodate outcome variables from various distribution families. For example, if *y*_*ijk*_ is continuous test scores and can be assumed to follow a normal distribution, an identity link function is used. If *y*_*ijk*_ is binary (e.g., pass/fail), a logit or probit link function can be used so that the linear predictor (which ranges from −∞ to ∞) is mapped to a 0 to 1 scale.

On the right-hand side of Equation (1), **X**_*ijk*_ is a covariate matrix, each row of which corresponds to a student and each column of which corresponds to a covariate, and ***β*** is a vector of regression coefficients for **X**_*ijk*_. The elements of the first column of **X**_*ijk*_ are 1 when the first element of ***β*** is the intercept. With no covariates and continuous math scores *y*_*ijk*_, the intercept is the grand mean, representing the *average* math scores of *average* students who attend an *average* school and lives in an *average* neighborhood.

In Equation (1), *u*_*j*_ and *u*_*k*_ are respectively the random effects associated with the two level-2 crossed factors, schools and neighborhoods, and are assumed to be normally distributed as $u_{j}\sim N\left(0,\sigma_{\text{school}}^{2}\right)$ and $u_{k}\sim N\left(0,\sigma_{\text{neighborhood}}^{2}\right)$. Additional technical details on CCREMs can be found in comprehensive review articles, such as Fielding and Goldstein (2006) and Cafri et al. (2015).

## 3. Software options

In this section, we provide a brief review of several existing software programs and packages for estimating CCREMs. These include HLM 7 (Raudenbush et al., 2011), MLwiN (Charlton et al., 2020), R package **lme4** (Bates et al., 2015), and functions in SAS 9.4 (SAS Inc, 2014). Although not discussed here to conserve space, additional software options are also available, such as Mplus (Muthén and Muthén, 1998–2017), SPSS (IBM Corp, 2020), and Bayesian options such as WinBUGS (Lunn et al., 2000) and Stan (Carpenter et al., 2017).

Subsequently, we describe the R package **PLmixed** (Jeon and Rockwood, 2018), which is selected for illustration in the present article. **PLmixed** can be downloaded from CRAN (https://cran.r-project.org/web/packages/PLmixed/).

### 3.1. Existing programs and packages

#### 3.1.1. HLM 7

HLM 7 (Raudenbush et al., 2011) is a commonly-used software program for fitting multilevel models. HLM 7 can fit two- and three-level CCREMs, using maximum likelihood estimation through the expectation–maximization (EM) algorithm (Raudenbush, 1993). For discrete outcome variables, HLM 7 estimates the models using the higher-order Laplace approximation. However, HLM 7 has limited capabilities in estimating CCREMs with more than three levels.

##### 3.1.1.1. MLwiN

MLwiN (Charlton et al., 2020) is another widely-used statistical software program for fitting various types of multilevel models. MLwiN can handle both continuous and discrete outcome variables. MLwiN also allows for inconstant level-1 variance (i.e., heteroscedasticity). MLwiN implements the iterative generalized least squares (IGLS) and Markov chain Monte Carlo (MCMC) estimation procedures (Browne, 2015). Estimating CCREMs with IGLS can be computationally intensive. Thus, splitting the data into separate groups is recommended to reduce the amount of storage needed. For example, in the school and neighborhood example, if some schools only have students from certain neighborhoods, and the students from these neighborhoods do not go to other schools, these schools and neighborhoods can be treated as separate groups. However, using this trick may not always be possible in all data situations. Using the MCMC option might not be an easy route either, as fundamental knowledge about the Bayesian method would be required for proper usage of the option.

##### 3.1.1.2. R package **lme4**

**lme4** (Bates et al., 2015) is a powerful R package for maximum likelihood estimation of linear and generalized linear mixed models. For non-continuous outcome variables, **lme4** utilizes the (penalized) iteratively reweighted least squares using the Laplace approximation (Doran et al., 2007). **lme4** is highly flexible, allowing for many nested and crossed random effects at multiple levels. **lme4** has also been used to estimate one-parameter logistic (1PL) item response theory (IRT) models (e.g., Doran et al., 2007; De Boeck et al., 2011), and their various extensions, such as IRTree models (e.g., De Boeck and Partchev, 2012). However, **lme4** cannot estimate models that involve factor loadings, such as two-parameter IRT models and factor analysis models. **blme** (Dorie and Dorie, 2015), a Bayesian version of **lme4**, offers additional flexibility, but estimating complex CCREMs with factor loading structures may still be challenging with this package.

##### 3.1.1.3. SAS 9.4

SAS 9.4 (SAS Inc, 2014) is a general-purpose statistical software program and it offers powerful procedures, such as GLIMMIX and MIXED, for fitting mixed models. GLIMMIX subsumes MIXED since it fits generalized linear mixed models, of which linear mixed models are a special case. GLIMMIX allows for accommodating complex nested and crossed random effects structures. GLIMMIX employs the restricted pseudo-likelihood (RPL) estimation (Wolfinger and O'connell, 1993), a linearization-based method, as its default estimation method for models with random effects. The PRL estimation method may yield biased parameter estimates especially for binary data. GLIMMIX also offers two integral approximation-based estimation methods, the Laplace approximation and adaptive Gauss-Hermite quadrature, but the latter may be computationally inefficient for estimating complex CCREMs.

### 3.2. R package **PLmixed**

**PLmixed** extends the **lme4** package to estimate extended generalized linear mixed models with factor structures. **PLmixed** implements a profile maximum likelihood estimation approach (Jeon and Rabe-Hesketh, 2012), taking advantage of the **lme4** estimation and the optim function (Byrd et al., 1995). **PLmixed** follows **lme4**'s syntax structure, and therefore, minimum learning may be required to utilize **PLmixed** for users who are already familiar with **lme4**.

To illustrate what **PLmixed** can do, let us return to the example used in Section 2.1. Suppose the researcher has tracked the students at multiple time points, and is interested in how the effects of schools change over time. For simplicity, assume the students did not change their school membership and residential areas (neighborhoods) during this time. In this longitudinal measurement context, we can extend the CCREM specified in Equation (1) as follows:

(2) $$\begin{array}{l} g\left(E\left(y_{itjk}|u_{jt},u_{k}\right)\right)={\text{X}}_{itjk}\beta +{\text{λ}}_{t}u_{jt}+u_{k}, \end{array}$$

where *y*_*itjk*_ indicates the *i*-th student's performance measure at time *t* (*t* = 1, …, *T*) in school *j* and neighborhood *k*. Here the new parameter λ_*t*_ is introduced to model the weight of the school random effect *u*_*jt*_ at time *t*. The weight parameters $\text{λ}={\left({\text{λ}}_{1},\ldots ,{\text{λ}}_{t},\ldots ,{\text{λ}}_{T}\right)}^{\prime }$ tell us how the impacts of schools on the students' math performance change over time. For identification purpose, one of the weights is fixed at 1 as a reference point (e.g., λ_1_ = 1). Since the weight parameter λ_*t*_ is multiplied by random effect *u*_*jt*_ and indicates the size/direction of the relationship between the outcome measures across time and the random effect, they can be seen as factor loadings as in the factor analysis framework. For example, λ_*t*_ is the factor loading of the random effect *u*_*jt*_ (or latent variable or factor) on the *t*-th outcome variable *y*_*itjk*_. In IRT, factor loadings are also called *item slopes* or *item discrimination parameters*. These factor loadings are not estimable with **lme4**.

The main function of the **PLmixed** package is PLmixed. The basic arguments of PLmixed function are identical to **lme4**'s glmer function's main arguments: formula (a two-sided linear formula that describes the model), data (a data frame containing variables in formula), and family (a GLMM family to specify the distribution of response variable). To estimate factor loadings, three new arguments are introduced: load.var (a vector of variables correspond to lambda and factor), lambda (the factor loading matrix), and factor (a list of factor names in lambda). We will further illustrate the usage of the PLmixed function in the following section through several CCREM applications. Of note, Rockwood and Jeon (2019) demonstrated the applications of **PLmixed** in the context of complex measurement and growth models, providing additional technical details of the profile likelihood estimation. We refer interested readers to Rockwood and Jeon (2019) for additional information.

## 4. Applications

In this section, we introduce several applications of CCREMs. We will first present several measurement models and methods as special applications of CCREMs. We will then introduce a couple of interesting CCREM applications that may be less well-known in applied research. For each of the applications, we first give a brief summary of the discussed models or methods. We then describe how to fit the models using **PLmixed** with an example dataset and briefly discuss the estimated results.

### 4.1. Measurement models and methods

#### 4.1.1. Random item-effect IRT models

##### 4.1.1.1. Background

IRT models describe the relationship between categorical item responses and the latent variable(s) to be measured with the test. The one-parameter logistic (1PL) model, also known as the Rasch model (Rasch, 1960), can be written for binary response *y*_*ij*_ to item *i* given by person *j* as follows:

(3) $$\begin{array}{l} \text{logit}\left(P\left(y_{ij}=1|\theta_{j}\right)\right)=\beta_{i}+\theta_{j}, \end{array}$$

where θ_*j*_ indicates person *j*'s level on the latent variable or construct of interest; respondents are typically assumed drawn from a population distribution, assuming $\theta_{j}\sim N\left(0,\sigma^{2}\right)$. β_*i*_ is the item intercept parameter representing the easiness level of item *i*. The 1PL model can also be seen as a two-level multilevel model with item responses at level 1 and respondents at level 2, where θ_*j*_ indicates person random effects and β_*i*_ indicates item fixed effects. The 1PL model can therefore be estimated with multilevel modeling packages, such as the R package **lme4** (Bates et al., 2015).

The 1PL model is extended to the two-parameter logistic (2PL) model by additionally accommodating the item slopes, or factor loadings α_*i*_:

(4) $$\begin{array}{l} \text{logit}\left(P\left(y_{ij}=1|\theta_{j}\right)\right)=\beta_{i}+\alpha_{i}\theta_{j}, \end{array}$$

The item slope parameters α_*i*_ are multiplied by the latent variable $\theta_{j}\sim N\left(0,\sigma^{2}\right)$, similar to Equation (2). For identifiability, one of the item factor loadings are fixed at 1 (or σ = 1). The 2PL model can be seen as a two-level multilevel model with a factor structure. Thus, **PLmixed** can estimate this model, while **lme4** cannot.

Van den Noortgate et al. (2003) presented an extended 1PL IRT model that assumes both respondents and items are random samples from the respective population distributions, therefore considered as random effects.

This so-called, random item-effect IRT model can be written as follows:

(5) $$\begin{array}{l} \text{logit}\left(P\left(y_{ij}=1|\theta_{j},\delta_{i}\right)\right)=\beta_{0}+\theta_{j}+\delta_{i}, \end{array}$$

where $\theta_{j}\sim N\left(0,\sigma^{2}\right)$ and $\delta_{i}\sim N\left(0,\tau^{2}\right)$ represent the respondent and item random effects, respectively, and β_0_ indicates the intercept. In the sense that the same set of items are given to respondents, item responses are cross-classified by respondents and items. When both respondents and items are considered random effects, as in Equation (5), the model can be seen as a CCREM with no covariates.

Under the CCREM framework, the random item-effect IRT model described above can be easily extended with person and item covariates. For example,

(6) $$\begin{array}{l} \text{logit}\left(P\left(y_{ij}=1|\theta_{j},\delta_{i}\right)\right)=\beta_{0}+{\text{x}}_{j}^{\text{P}}\beta^{\text{P}}+{\text{x}}_{i}^{\text{I}}\beta^{\text{I}}+{\text{x}}_{ij}^{\text{P,I}}\beta^{\text{P,I}}+\theta_{j}+\delta_{i}, \end{array}$$

where ***β***^P^ is a vector of regression coefficients for ${\text{x}}_{j}^{\text{P}}$, a vector of person *j*'s characteristics (e.g., gender and motivation level). ***β***^I^ is a regression coefficient vector for ${\text{x}}_{i}^{\text{I}}$, a vector of item *i*'s features (e.g., item length and cognitive complexity). ***β***^P,I^ is a vector of regression coefficients of ${\text{x}}_{ij}^{\text{P,I}}$, which is a vector of person-by-item interactions (e.g., whether person *i*'s background, such as gender, interacts with item *i* or item *i*'s feature). In addition, the CCREM framework allows for further expansions of the random item-effect IRT model with more complex person and item clustering structures; for example, respondents and/items are nested or cross-classified with higher-level units (e.g., respondents are cross-classified with schools and neighborhoods, and items are cross-classified with two or more cognitive functions).

##### 4.1.1.2. Illustration

To show how to fit a random item-effect IRT model with **PLmixed**, we use the simulated data IRTsim in the package. The installation of the package and the first six rows of the data are presented below.


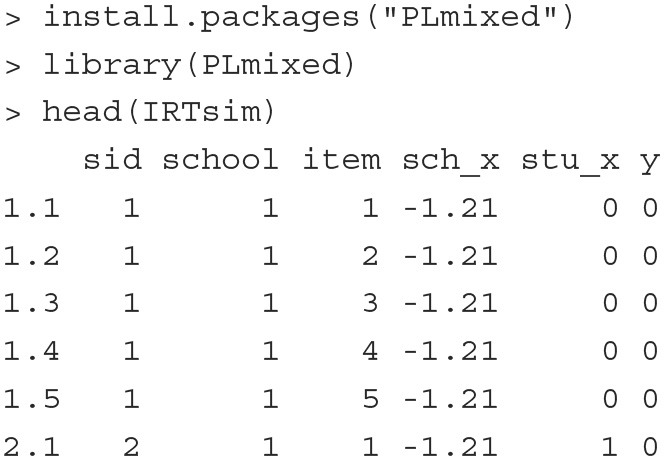


The data contain 6 variables and 2,500 binary item responses from a cognitive test. The first three columns of the data are sid, school, and item, indicating student ID, school ID, and item ID, respectively. There are a total of 500 students from 26 school and 5 items. sch_x represents a continuous school-level covariate. stu_x is a binary student-level covariate. y indicates students' binary responses that takes the value of 1 if the answer is correct and 0 otherwise.

**Model** To illustrate, we specify a random item-effect IRT model with a cross-classified structure of respondents with schools. We include student-level and school-level covariates to examine their impacts on the success probability.

(7) $$\begin{array}{l} \text{logit}(P(y_{ijk}=1|\theta_{jk},\theta_{k},\delta_{i}))=\beta_{0}+\beta_{1}\times {\text{sch\_x}}_{k}+\beta_{2}\\ \;\times {\text{stu\_x}}_{jk}+\delta_{i}+\theta_{jk}+\theta_{k}, \end{array}$$

where β_0_ is the intercept, β_1_ is the regression coefficient for the school-level covariate, β_2_ is the regression coefficient for the student-level covariate. $\delta_{i}\sim N\left(0,\tau^{2}\right)$ is the random item effect, where τ indicates how much the item easiness varies across the test items. $\theta_{jk}\sim N\left(0,\sigma_{\text{stu}}^{2}\right)$ and $\theta_{k}\sim N\left(0,\sigma_{\text{sch}}^{2}\right)$ represent the student and school random effects, respectively.

**Fitting the model** The model can be fitted with the below **PLmixed** syntax:


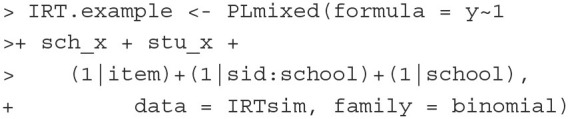


The argument formula follows Equation (7). The item response y is a function of three fixed-effect and three random-effect terms. The inclusion of the term 1 indicates that the intercept of the linear predictor β_0_ is estimated. With sch_x and stu_x, the regression coefficients, β_1_ and β_2_, are estimated as well. (1|item), (1|sid:school) and (1|school) are the three random-effect terms. The colon between sid and school is used to indicate that the students are nested within schools. Here, the argument family = binomial means that the binomial family with a logit link function is used. Other than the default logit link, a probit link function can also be used with the syntax family = binomial(link="probit"). All results are saved in an object named IRT.example.

After fitting the model, the estimated results are summarized with the summary() function.


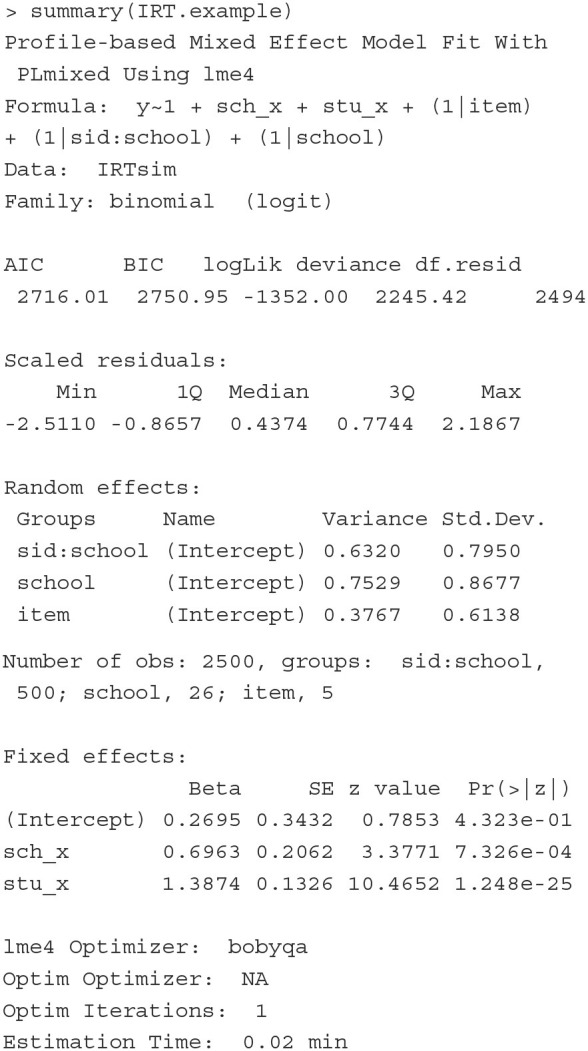


The first section of the summary echos the formula we specified, the data, and the link function. The second section presents model fit indices including the AIC, BIC, and a summary of the scaled residuals.

In the Random effects section, the estimates of variances and standard deviations of the three random effects are presented. The last line of this section lists the numbers of observations, students, schools and items. These estimates indicate that about 42.7% (= 0.753/ [0.632 + 0.753 + 0.377]), 35.8% (= 0.632/ [0.632 + 0.753 + 0.377]), and 21.4% (= 0.377/ [0.632 + 0.753 + 0.377]) of the variation in the data are explained by the differences between schools, between students, and between items, respectively.

In the Fixed effects section, the estimates of the fixed effects are presented. The intercept estimate is 0.2695, indicating that the probability that the an *average* student (i.e., θ_*jk*_ = 0) in the stu_x=0 group from an *average* school (i.e., θ_*k*_ = 0) with sch_x=0 correctly answers an *average* item (i.e., δ_*i*_ = 0) is about 0.577 (= 1/ [1+exp(–0.2695)]). The change in the logit that is associated with one unit increase in sch_x is 0.6963, and the corresponding standard error (SE) is 0.2062. The average difference between the stu_x = 0 group and stu_x = 1 group is 1.3874, with SE of 0.1326.

The last section of the result lists lme4 optimizer, optim optimizer, the number of optim iterations, and the estimation time.

#### 4.1.2. Rater effect models

##### 4.1.2.1. Background

Social science studies often utilize assessment by raters. In this case, raters contribute to the variance in the assessment data, and this variance due to rater effects is not related to the variation of the ratees in their performance (Scullen et al., 2000). Since rating data are cross-classified by raters and ratees, CCREMs are a natural option to address rater effects. For example, CCREMs have been used to estimate rater effects in cross-sectional data (e.g., Jayasinghe et al., 2003; Murphy and Beretvas, 2015; Lei et al., 2018; Martinez et al., 2020; Chen et al., 2021) and in longitudinal data settings (e.g., Guo and Bollen, 2013). Under the CCREM framework, researchers can differentiate rater variance from other sources of variances, and incorporate covariates to answer useful research questions. For example, a large rater variance indicates that ratings vary to a large degree across raters, implying that rater training needs to focus on improving consistency across raters (by making the scoring rubrics tighter and stricter, for instance). With covariates, one can find out whether particular characteristics of raters, ratees, and rater-and-ratee combinations may be associated with systematically higher or lower ratings.

##### 4.1.2.2. Illustration

To show how to fit a rater effect model as a CCREM, we first generate a dataset that mimics the empirical data analyzed by Jayasinghe et al. (2003). The simulated data include 2,401 evaluations of 1,580 assessors on 673 grant proposals. Each assessor and proposal belongs to one of the 28 fields of study, which can be further categorized into nine general discipline panels, such as Physics and Mathematics. The first six rows of the simulated dataset are printed below.


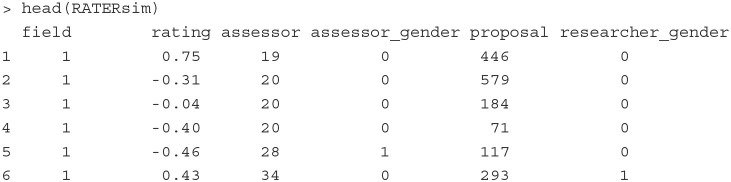


The outcome variable is the rating (rating) of a proposal (proposal) provided by an assessor (assessor) in a field of study (field). The mean and standard deviation of the ratings are 0.14 and 1.05, respectively. The number of proposals assessed by each assessor ranges from 1 to 3. The number of ratings each proposal receives ranges from 2 to 7. The numbers of assessors, proposals and ratings in the fields range from 15 to 83, from 12 to 33, and from 37 to 122, respectively. Other covariates included in the data are the gender of the assessors (assessor_gender) and the gender of the first author of the proposals (researcher_gender). For these gender variables, 0 indicates the assessor/author is a male, and 1 indicates the assessor/author is a female.

**Model** We specify a three-level CCREM where ratings (at level 1) are cross-classified by assessors (raters) and proposals (at level 2) both of which are nested within fields (at level 3). In addition to the random effects of assessors, proposals, and fields, we examine the effects of the genders of assessors, first authors of proposals, and their interactions. The rater effect CCREM can then be specified as follows:

(8) $$\begin{array}{l} y_{ijk}|u_{ik},u_{jk},u_{k}=\beta_{0}+\beta_{1}x_{ik}^{\text{R}}+\beta_{2}x_{jk}^{\text{P}}+\beta_{3}x_{ijk}^{\text{R,P}}+u_{ik}+u_{jk}+u_{k}+\varepsilon_{ijk}, \end{array}$$

where *y*_*ijk*_ is the rating of proposal *j* in field *k* assessor *i* provides. $x_{ik}^{\text{R}}$, $x_{jk}^{\text{P}}$ and $x_{ijk}^{\text{R,P}}$ are the assessor' gender (1: female; 0: male), first author's gender (1: female; 0: male), and the interaction between the two (1: the assessor and author are both females; 0: the assessor and author are of different genders or are both males), respectively. β_0_ is the intercept and β_1_ to β_3_ are the regression coefficients for the three gender-related covariates. The regression coefficient β_3_ of the interaction term can answer an interesting question that whether female assessors' rating on the proposals written by female authors are consistently higher or lower. The assessor, proposal and field random effects are assumed $u_{ik}\sim N\left(0,\sigma_{\text{assessor}}^{2}\right)$, $u_{jk}\sim N\left(0,\sigma_{\text{proposal}}^{2}\right)$, and $u_{ijk}\sim N\left(0,\sigma_{\text{field}}^{2}\right)$.

**Fitting the model** The below PLmixed syntax can be used to fit the model specified in Equation (8).


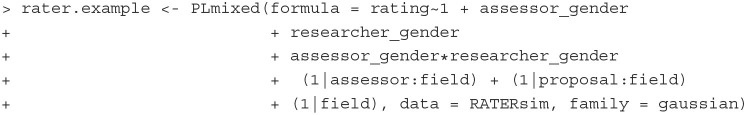


This syntax is similar in structure to the syntax shown in Section 4.1.1. Thus, we omit giving all specific details of the arguments. The argument family = gaussian is used since the response data are continuous in this example. If family is omitted, Gaussian family with an identity link function is applied by default.

The summary() function gives the summary of the estimated results. To save space, we print here only the parameter estimates and the associated standard errors in the Random effects and Fixed effects sections.


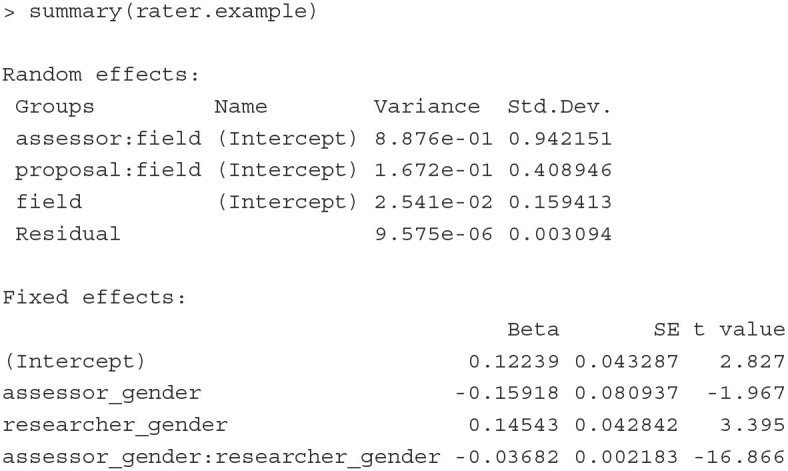


In the Random effects section, the estimates of variances are shown. Consistent with the results shown in Jayasinghe et al. (2003), assessors explain about 82% (= 0.8876/ [0.8876+0.1672+0.0254]) of the total variation in ratings, proposals explain about 15% (= 0.1672/ [0.8876+0.1672+0.0254]), and fields explain 3% (= 0.0254/ [0.8876+0.1672+0.0254]) of the variation in the data. The large between-assessor variance tells us that improving consistency in ratings across assessors would be a useful consideration to improve the proposal assessment system.

In the Fixed effects section, the estimates of regression coefficients and the associated standard errors are presented. The results suggest that female assessors on average provide lower ratings than male assessors, female authors on average receive higher ratings than male authors, and proposals written by female authors receive lower ratings from female assessors.

#### 4.1.3. Multitrait-multimethod models

##### 4.1.3.1. Background

The multitrait-multimethod (MTMM) (Campbell and Fiske, 1959) models have been applied broadly in applied social science research (e.g., Lance and Sloan, 1993; Biesanz and West, 2004; DeYoung, 2006). When multiple traits (e.g., the Big Five) are measured with multiple methods (e.g., self report or peer report), MTMM models are used to differentiate method effects from trait effects. MTMM analysis helps researchers evaluate convergent and discriminant validity of the main measures; the methods designed to measure the same traits should be highly correlated, while the traits should not correlate highly with the methods.

The flexible CCREM framework allows researchers to extend the classical MTMM model in a variety of ways, e.g. by adding covariates and dealing with multilevel/cross-classified data structures. These extended models can be easily estimated with a CCREM package of choice, such as **PLmixed**, without needing to develop a new estimation routine.

##### 4.1.3.2. Illustration

To show how the MTMM model can be estimated as a CCREM, we simulate a dataset that reproduces the MTMM covariance matrix reported by DeYoung (2006). The simulated data include continuous scores on the Big Five obtained from subjects (*n* = 500) and three of their peers. Each subject has 20 scores, where five are self-reported and the other 15 are reported by three peers (five per peer). The five self-reported scores are measures 1–5, and the 15 peer-reported scores are measures 6–10. The first six row of the simulated data are printed below.


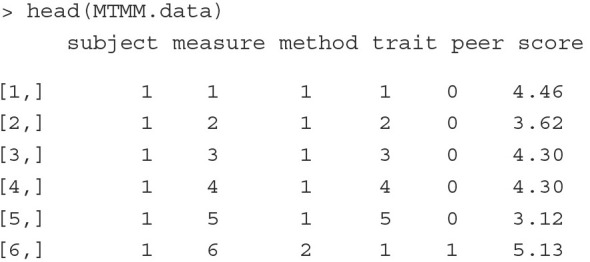


The variable subject, measure, method, trait and peer are indicators of subject, scale, method (1: self report, 2: peer report), trait (1 = *Extraversion*, 2 = *Agreeableness*, 3 = *Conscientiousness*, 4 = *Neuroticism*, 5 = *Openness/Intellect*) and peer (0: self report). The last column, score, is the outcome variable.

**Figure 1:**
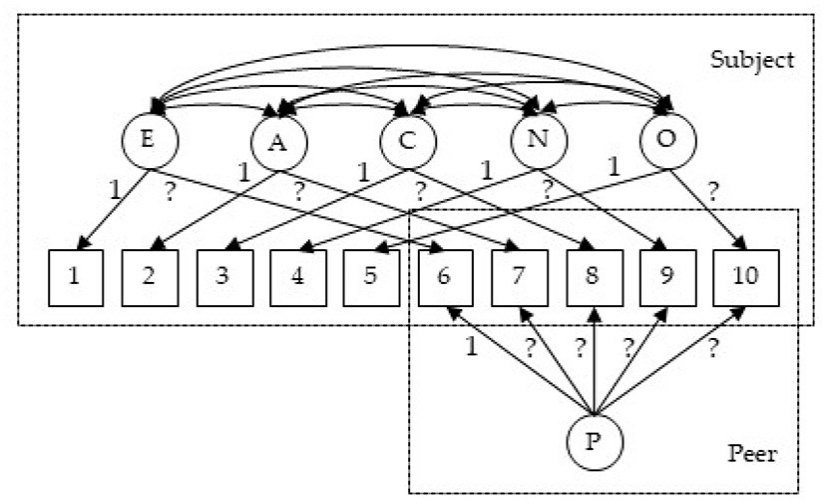
Circles in the figure represent latent variables: E, Extraversion; A, Agreeableness; C, Conscientiousness; N, Neuroticism; O, Openness/Intellect; P, peer rater effect. Squares are observed ratings: measures 1–5 are self-reported scores and measures 6–10 are peer-reported. The 1 in the figure represents fixed loading (imposed for identification purposes). The ? represents a loading that needs to be estimated.

**Model** Figure 1 illustrates the MTMM model that we consider for this dataset. Mathematically, the model is specified as follows:

(9) $$\begin{array}{l} y_{itpj}|\theta_{tj}^{T},\theta_{p}^{M}=\beta_{i}+{\text{λ}}_{it}^{T}\theta_{tj}^{T}+{\text{λ}}_{ip}^{M}\theta_{p}^{M}+\varepsilon_{itpj}, \end{array}$$

where *y*_*itpj*_ represents peer *p*'s rating to measure *i* on trait *t* of subject *j*. β_*i*_ denotes the intercept of measure *i*, ${\text{λ}}_{it}^{T}$ indicates measure *i*'s loading on $\theta_{tj}^{T}$ for *t*-th trait, and ${\text{λ}}_{ip}^{M}$ indicates measure *i*'s loading on $\theta_{p}^{M}$, the peer rater effect. The trait latent variables and peer/rater latent variables are assumed: $\theta_{j}^{T}\sim N\left(0,{\text{Σ}}_{\text{T}}\right)$ and $\theta_{p}^{M}\sim N\left(0,\sigma_{M}^{2}\right)$. The residual term is assumed $\varepsilon_{itpj}\sim N\left(0,\sigma_{e}^{2}\right)$.

**Fitting the model** To fit the above model with PLmixed, we first specify the factor loading lambda matrix according to the diagram (Figure 1). Rows of the lambda matrix correspond to the measures, and columns correspond to the latent variables. For identification purpose, we fix the first loading of each lambda matrix to 1. Note that as measures 6–10 are peer-reported, the first five elements of the rater effect latent variable (i.e., the last column of the lambda matrix) are zeros. NA represents unknown parameters that are to be estimated.


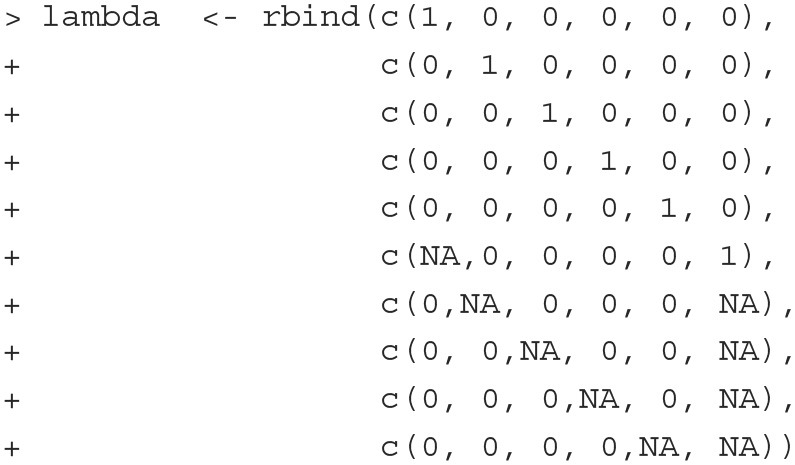


The full PLmixed syntax can be specified as follows:


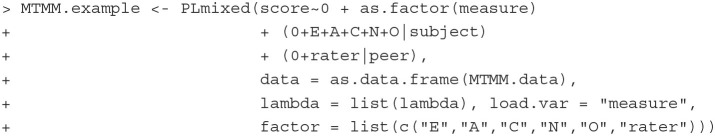


The 0 is included to ensure no intercept is estimated. We use as.factor(measures) to estimate measure-specific intercepts. The five trait latent variables are named E, A, C, N and O. As assumed, these latent variables are correlated and vary across subjects. If independence is assumed among the trait latent variables, the syntax would be specified as (E|subject) + (A|subject) + (C|subject) + (N|subject) + (O|subject). The last term in the formula argument corresponds to the random rater effects. The lambda argument indicates the factor loading matrix we specified earlier. The load.var argument indicates the variable that defines the factor loading matrix. The factor shows the names of the latent variables.

Below shows the summary of the estimated results.


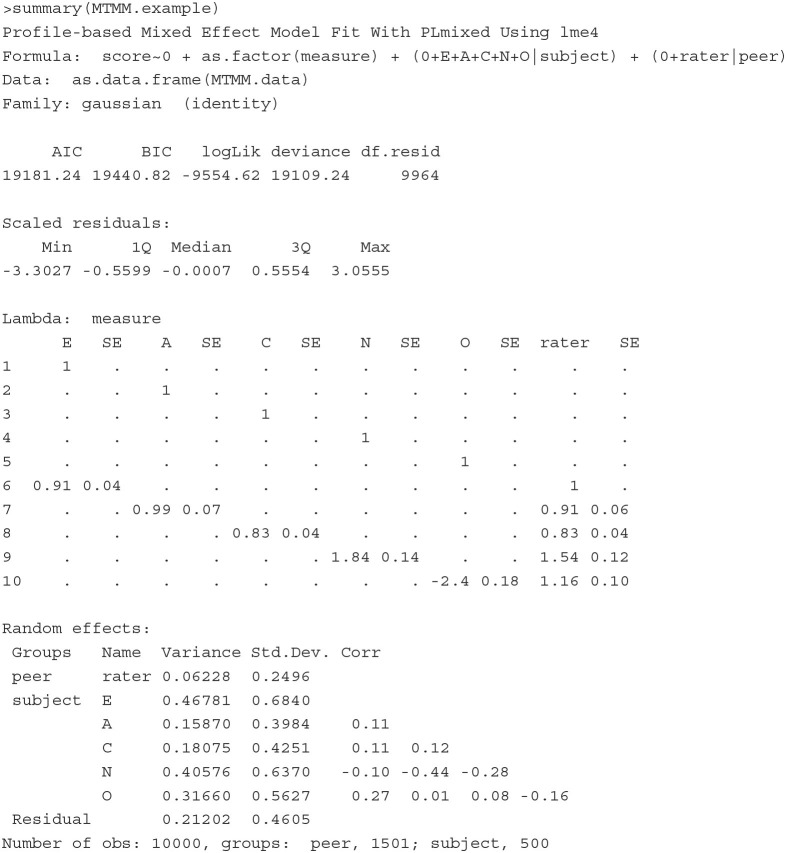



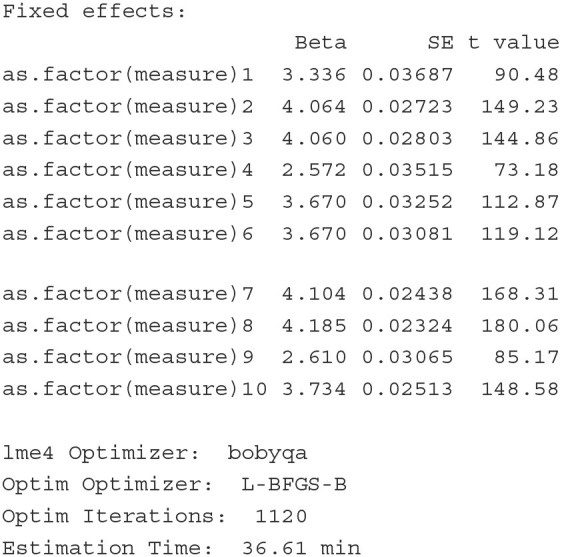


The first section of the results echos the formula, and the second section shows model fit indices. The Lambda section shows the estimates of factor loadings and the corresponding standard errors. The negative loading of measure 10 on the latent variable O indicates that the peer-reported scores are negatively associated with *Openness/Intellect*, suggesting that these peer-reported scores of measure 10 should be closely examined as they do not seem to properly measure the target latent variable *Openness/Intellect*. Following the factor loading section, the Random effects section lists the estimates of variances of all latent variables, and the correlations of the five traits. In the Fixed effects section, measure-specific intercepts are presented. In the last section, information about **lme4** optimizer is listed.

#### 4.1.4. Generalizability theory

##### 4.1.4.1. Background

Generalizability theory (G-theory) is a statistical framework for evaluating the generalizability (or reliability) of measurements (Shavelson and Webb, 1991). In G-theory, observed scores are decomposed into additive effects of multiple facets (i.e., sources of variations). If levels of a facet in a measurement design are viewed as random samples from the universe of all levels, or if researchers would like to generalize beyond these observed levels, this facet is a *random* facet. In contrast, if all possible levels of a facet are included in the design, or if there is no need for generalization, this facet is treated as a *fixed* facet. G-theory includes two types of studies, the generalizability study (G study) and the decision study (D study). In a G study, variances associated with the facets are computed. In a D study, different generalizability coefficients are constructed, using the variance estimates computed from the G study. A generalizability coefficient is analogous to a reliability coefficient, while it depends on which facets are considered random.

Vangeneugden et al. (2005) utilized a linear mixed model framework to estimate G-theory models, so that different kinds of generalizability/reliability coefficients can be derived from the estimates of variances. Choi (2013) and Choi and Wilson (2018) presented a generalized linear latent and mixed modeling approach (GLLAMM) (Skrondal and Rabe-Hesketh, 2004) to combine G-theory and IRT. Jiang (2018) and Jiang et al. (2020) showed how generalizability variance components from univariate and multivariate generalizability theory can be estimated within the linear mixed model framework with general-purpose R packages.

##### 4.1.4.2. Illustration

To illustrate how a G-theory model can be estimated and extended as a CCREM, we analyze the Brennan.3.2 dataset from the **gtheory** R package (Moore, 2016). The dataset contains scores of 10 persons' performance on 3 tasks, each of which is rated by 4 raters (i.e., the classical person × [rater:task] design). The first six rows of the data are printed below.


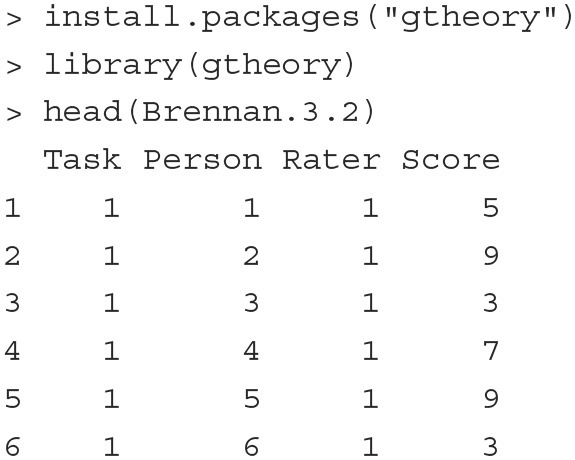


The outcome variable is the score (Score), which ranges from 1 to 9. The 10 persons (Person) complete all three tasks (Task). Raters (Rater) 1 to 4 assess task 1, raters 5 to 8 assess task 2, and raters 9 to 12 assess task 3.

**Model** In this person × (rater:task) design, there are five sources of variation: persons, tasks, raters, person-task interactions and residuals. The data show a cross-classified structure where the scores are cross-classified by persons and raters, which are nested within tasks. We specify the G-theory model as a CCREM as follows:

(10) $$\begin{array}{l} y_{ijk}|u_{jk},u_{k},u_{ik}=\beta_{0}+u_{i}+u_{jk}+u_{k}+u_{ik}+\varepsilon_{ijk}, \end{array}$$

where *y*_*ijk*_ is person *i*'s score on task *k* by rater *j*, $u_{i}\sim N\left(0,\sigma_{\text{person}}^{2}\right)$ represents the person random effect, $u_{jk}\sim N\left(0,\sigma_{\text{rater}}^{2}\right)$ is the rater random effect, $u_{k}\sim N\left(0,\sigma_{\text{task}}^{2}\right)$ is the task random effect, $u_{ik}\sim N\left(0,\sigma_{\text{person}\times \text{task}}^{2}\right)$ is the person-task interaction random effect, and $\varepsilon_{ijk}\sim N\left(0,\sigma_{\text{e}}^{2}\right)$ is the residual term.

**Fitting the model** The PLmixed syntax to fit this model is displayed below.


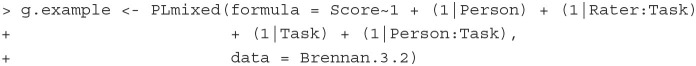


The summary of the estimated results is shown below; only the Random effects and Fixed effects sections are listed to conserve space.


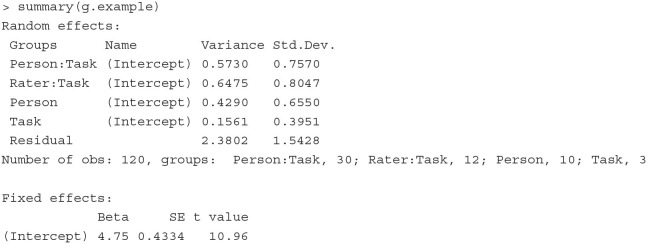


With the estimates of variances associated with multiple variance sources, we can derive desired generalizability/reliability coefficients. For example, if we want to generalize persons' scores over raters and tasks, a generalizability coefficient is constructed as:

$$\begin{array}{l} R_{\rho^{2}\text{Rel}}=\frac{\sigma_{\text{person}}^{2}}{\sigma_{\text{person}}^{2}+\sigma_{\text{rater}}^{2}+\sigma_{\text{task}}^{2}+\sigma_{\text{person*task}}^{2}+\sigma_{\text{e}}^{2}}\\ \;=\frac{0.5730}{0.5730+0.6475+0.4290+0.1561+2.3802}=0.14. \end{array}$$

The reliability coefficient above is fairly low (consistent with the result from the **gtheory** package). This means that it would be difficult to generalize subjects' scores over different raters and tasks, calling for further investigation on the assessment data.

### 4.2. Other applications

#### 4.2.1. Social network analysis

##### 4.2.1.1. Background

Social network analysis (SNA) examines how individuals are connected and how social connections influence individuals and their behaviors (Otte and Rousseau, 2002). A number of methods and techniques have been developed for social network analysis, such as stochastic actor oriented models (SAOM) (e.g., Snijders, 1996, 2017), exponential random graph models (ERGM) (e.g., Holland and Leinhardt, 1981; Frank and Strauss, 1986; Cranmer and Desmarais, 2011), and latent space models (e.g., Hoff et al., 2002; Sewell and Chen, 2015).

CCREMs have also been utilized to study social network connections and dependencies among individuals generated due to their social networks. For example, Tranmer et al. (2014) considered individuals' ego-nets (of size 2 and 3) as their classification structures and applied a CCREM to account for the dependencies due to the network structure. They found that ignoring network dependencies as such would bias the estimates of fixed and random effect parameters in the model. De Nooy (2011) analyzed reviews/interviews among 40 literary authors and critics in the Netherlands during 1970–1980. The network model adopted in this study can be viewed as a CCREM in the sense that the model viewed review/interviews were cross-classified by authors and critics. Koster et al. (2015) showed that the multilevel formulation of the Social Relations Model (SRM) (Snijders and Kenny, 1999) could be estimated as a cross-classified Poisson model. The SRM as a CCREM partitions the total variance in social network data into variance components contributed by multiple sources.

##### 4.2.1.2. Illustration

To illustrate how a CCREM can be used for social network analysis, we analyze a dyadic network dataset used by Koster et al. (2015). The data were collected in a village of indigenous Ye'kwana horticulturalists with eight households in Venezuela. These eight households lead to 28 dyads. The first six rows of the data are printed below:


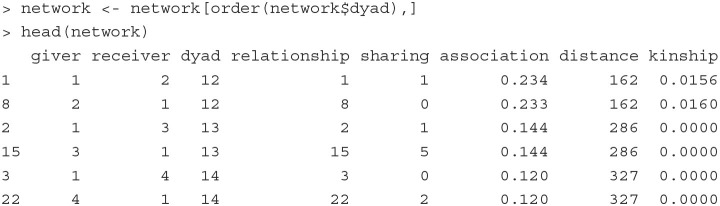


The outcome variable is the total number of meals (sharing) provided from one household (giver) to another (receiver). Each giver-receiver pair defines a relationship (relationship). Note the relationship variable is directed, meaning that the values may not be the same for household A-B and B-A pairs. Other covariates are the distance between households (distance), the genetic relatedness between households (kinship), and an association index which is a measure of interactions between each pair of households (association).

**Model** The number of meals *y*_*ij*_ that household *i* gives to household *j* is assumed to follow a Poisson distribution with mean μ_*ij*_, i.e., *y*_*ij*_ ~ Pois(μ_*ij*_). For simplicity, we consider no covariates and specify a Poisson CCREM as follows:

(11) $$\begin{array}{l} \text{log}\left(\mu_{ij}\right)=\beta_{0}+g_{i}+r_{j}+u_{\left|ij\right|}+\varepsilon_{ij}, \end{array}$$

where β_0_ is the intercept, *g*_*i*_ and *r*_*j*_ represent the giver and receiver random effects, respectively. The two random effects are assumed to be independent with $g_{i}\sim N\left(0,\sigma_{\text{giver}}^{2}\right)$ and $r_{j}\sim N\left(0,\sigma_{\text{receiver}}^{2}\right)$. We further decompose the relationship random effects into two parts: *u*_|*ij*|_ represents the symmetric (undirected) relationship random effect, with $u_{\left|ij\right|}\sim N\left(0,\sigma_{\text{dyad}}^{2}\right)$. The |·| is used in the subscript to indicate that this effect is symmetric, meaning that it takes the same value within each dyad. *e*_*ij*_ is the asymmetric (directed) relationship random effect, with $\varepsilon_{ij}\sim N\left(0,\sigma_{\text{relation}}^{2}\right)$. This effect is asymmetric, meaning that the value of *e*_*ij*_ can be different for each observation within a dyad. Covariates at the giver, receiver and relationship levels can be also incorporated into the model.

**Fitting the model** The network model described above can be fit with the below PLmixed syntax.


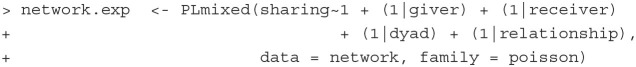


We show below the Random effects and Fixed effects sections of the estimated results.


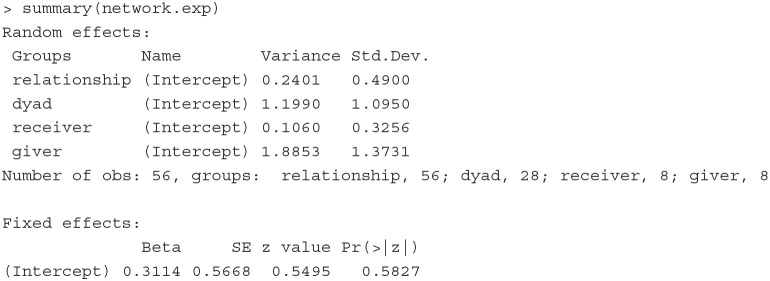


With the variance estimates of the random effects, we can quantify the contribution of each variation source to the total variance. For example, the amount of total variation attributed to the givers is 0.549 (= 1.8853/[0.2401 + 1.1990 + 0.1060 + 1.8853]); that is, givers explain 54.9% of the total variation in the data. In contrast, receivers explain only 3.1% of the variance (0.031 = 0.1060/[0.2401 + 1.1990 + 0.1060 + 1.8853]).

#### 4.2.2. Effects of country of origin and destination

##### 4.2.2.1. Background

Another interesting application of CCREMs is found in immigrant studies that examine impacts of countries of origin and destination on various outcomes of immigrants. Examples of such studies include Levels et al. (2008), Kislev (2017), and Dronkers et al. (2014).

##### 4.2.2.2. Illustration

For illustration, we simulate data that mimic the PISA data analyzed by Levels et al. (2008). The simulated data include the math scores of 7,403 immigrant students who were born in 35 different countries and took the PISA math test in 13 different countries. Below we print out the first six rows of the dataset.


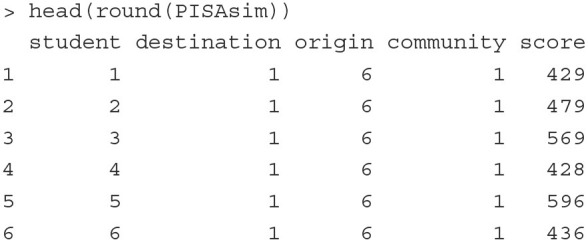


The outcome variable is the math scores (score) of a student (student) who was born in an origin country (origin) and took the PISA test in a destination country (destination). The mean and standard deviation of scores are 503 and 98, respectively. Each combination of the origin and the destination countries defines an immigrant community (community). Since not all origin countries are presented in all destination countries, the data contain 67 (instead of 13 × 35 = 455) different immigrant communities. The number of students in each of the communities ranges from 90 to 144.

**Model** In this example, students are cross-classified by countries of origin and destination. The communities indicate the origin-by-destination combinations. To differentiate the three types of contextual effects on students' math scores, we specify the following CCREM model:

(12) $$\begin{array}{l} y_{ijk}|u_{j},u_{k},u_{jk}=\beta_{0}+u_{j}+u_{k}+u_{jk}+\varepsilon_{ijk}, \end{array}$$

where *y*_*ijk*_ represents the math score of student *i*'s, who was born in country *j*, took the test in country *k* and lives in the immigrant community defined by the origin and destination countries. β_0_ is the intercept, $u_{j}\sim N\left(0,\sigma_{\text{origin}}^{2}\right)$ and $u_{k}\sim N\left(0,\sigma_{\text{destination}}^{2}\right)$ are the origin country and destination country random effects, respectively, and $u_{jk}\sim N\left(0,\sigma_{\text{community}}^{2}\right)$ is the community random effects, as specified as the cell effects (cells created by origin and destination country classifications). $\varepsilon_{ijk}\sim N\left(0,\sigma_{\text{e}}^{2}\right)$ is the residual term.

**Fitting the model** The above model can be estimated with the PLmixed syntax below:


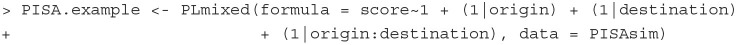


Elements in the formula argument correspond to the terms in Equation (12). The 1 represents the intercept, and (1|origin), (1|destination) and (1|origin:destination) are the three random-effect terms.

Below we display the Random effects and Fixed effects sections of the estimated results.


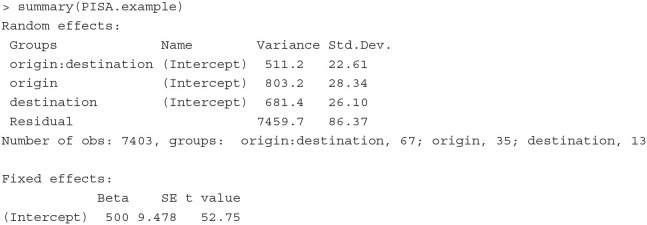


The Random effects section lists the variance estimates of random effects. Here origin:destination indicates the community random effects specified as the cell effects. The results suggest that the immigrant community, the country of origin, and the country of destination explain 5.4% (= 511.2/[511.2 + 803.2 + 681.4 + 7459.7]), 8.5% (= 803.2/[511.2 + 803.2 + 681.4 + 7459.7]) and 7.2% (= 681.4/[511.2 + 803.2 + 681.4 + 7459.7]) of the total variation in the math scores, respectively. It is interesting that the country of origin still has influence immigrant students' math scores, and the students' immigrant community shows as large influence as the destination county on immigrant students' math outcomes.

## 5. Concluding remarks

In this article, we aim to introduce some modern applications of CCREMs for social science studies. We presented some existing models and methods widely used in applied research as applications of CCREMs. These applications and their connections to CCREMs might have been shown in the literature by different authors in separate studies. However, to our best knowledge, these different applications have not been reviewed, discussed, and integrated together in a single paper.

We want to stress again the usefulness of understanding the connections between different models that serve for seemingly different purposes within the CCREM framework. As discussed earlier, those models can easily be extended to accommodate complex clustering and covariate structures in the data for any units of analysis. Further, the models presented in this paper can be integrated when desirable under the unifying framework that CCREM offers. For example, the rater effect model can be combined with the random item-effect IRT model, and/or with the multitrait-multimethod model, and/or the G-theory model. As another example, the model presented for a study of country of origin and destination may be extended with a measurement model such as the two-parameter IRT model. Without having these perspectives, researchers may need to develop their own estimation routines every time there is a need for such models, which can be inefficient, time consuming, and unnecessary.

It may be worth commenting that the flexibility and generality of the CCREM framework come with a cost in the sense that researchers need to understand the CCREM framework, and translate the general language of the CCREM framework into the specific modeling contexts of choice, which may not be an easy task. The current article is written to help remediate such a challenge by providing a practical guide to the CCREM and its applications.

Lastly, we chose the R package **PLmixed** to illustrate the estimation of the discussed models. **PLmixed** is a convenient choice as it is freely available and it can estimate all of the models presented in this paper and many of their combinations. Of note, when no factor structures are involved, the models presented in this paper can also be estimated with the R package **lme4**. But when factor loadings are included, **lme4** can no longer be used. In addition, an important future line of work is to compare **PLmixed** with other packages specialized for specific models as well as other general-purpose software programs in terms of the model specification and estimated results. Further, it would also be useful to conduct rigorous simulation studies to compare the performance accuracy of different software packages for estimating different CCREM applications introduced in this paper.

## Author's note

SH is currently an Assistant Professor at Indiana University Bloomington. MJ is an Associate Professor at the University of California, Los Angeles.

## Data availability statement

The original contributions presented in the study are included in the article/supplementary material, further inquiries can be directed to the corresponding author.

## Author contributions

SH and MJ drafted the manuscript. Both authors contributed to the article and approved the submitted version.

## Conflict of interest

The authors declare that the research was conducted in the absence of any commercial or financial relationships that could be construed as a potential conflict of interest.

## Publisher's note

All claims expressed in this article are solely those of the authors and do not necessarily represent those of their affiliated organizations, or those of the publisher, the editors and the reviewers. Any product that may be evaluated in this article, or claim that may be made by its manufacturer, is not guaranteed or endorsed by the publisher.

## Author disclaimer

Opinions are those of the authors alone and do not necessarily reflect those of the authors' home institutions.
